# Macronutrient and Micronutrient Intake during Pregnancy: An Overview of Recent Evidence

**DOI:** 10.3390/nu11020443

**Published:** 2019-02-20

**Authors:** Aya Mousa, Amreen Naqash, Siew Lim

**Affiliations:** 1 Monash Centre for Health Research and Implementation (MCHRI), School of Public Health and Preventive Medicine, Monash University, Melbourne VIC 3168, Australia; siew.lim1@monash.edu; 2 Department of Pharmaceutical Sciences, University of Kashmir, Srinagar, Jammu and Kashmir 190006, India; anaqash.scholar@kashmiruniversity.net

**Keywords:** nutrition, macronutrients, micronutrients, pregnancy, reproduction, maternal health, neonatal outcomes

## Abstract

Nutritional status during pregnancy can have a significant impact on maternal and neonatal health outcomes. Requirements for macronutrients such as energy and protein increase during pregnancy to maintain maternal homeostasis while supporting foetal growth. Energy restriction can limit gestational weight gain in women with obesity; however, there is insufficient evidence to support energy restriction during pregnancy. In undernourished women, balanced energy/protein supplementation may increase birthweight whereas high protein supplementation could have adverse effects on foetal growth. Modulating carbohydrate intake via a reduced glycaemic index or glycaemic load diet may prevent gestational diabetes and large-for-gestational-age infants. Certain micronutrients are also vital for improving pregnancy outcomes, including folic acid to prevent neural tube defects and iodine to prevent cretinism. Newly published studies support the use of calcium supplementation to prevent hypertensive disorders of pregnancy, particularly in women at high risk or with low dietary calcium intake. Although gaps in knowledge remain, research linking nutrition during pregnancy to maternofoetal outcomes has made dramatic advances over the last few years. In this review, we provide an overview of the most recent evidence pertaining to macronutrient and micronutrient requirements during pregnancy, the risks and consequences of deficiencies and the effects of supplementation on pregnancy outcomes.

## 1. Introduction

Pregnancy is a time of rapid and profound physiological changes from the time of conception until birth. Nutritional requirements increase during pregnancy to maintain maternal metabolism and tissue accretion while supporting foetal growth and development [1]. Poor dietary intakes or deficiencies in key macronutrients and micronutrients can therefore have a substantial impact on pregnancy outcomes and neonatal health. Increasing evidence suggests that the effects of foetal nutrition may persist well into adulthood, with possible intergenerational effects [2]. Although a healthy and varied diet remains the preferred means of meeting nutritional requirements, some nutritional needs in pregnancy are challenging to meet with diet alone. As such, supplement use may be prescribed and food fortification programs such as salt iodization, vitamin D-fortified milk and folate-fortified breads and cereals also play an important role in supporting women to meet the increased nutritional demands of pregnancy [3].

Despite a large body of evidence supporting the importance of adequate nutrition in pregnancy, around 20% to 30% of pregnant women worldwide suffer from some vitamin deficiency [1]. A number of studies and comprehensive meta-analyses have been published over the last few years linking nutritional intake or lack thereof, to maternofoetal outcomes; however, there remain several areas of uncertainty. Published systematic and narrative reviews examining nutrition in pregnancy have tended to focus primarily on micronutrients [3,4] or have examined specific nutrients in isolation such as carbohydrates [5], folic acid [6] or vitamin D [7] or specific outcomes such as birth defects [8] or pregnancy loss [9]. Many reviews describing macro- and micro-nutriture in relation to pregnancy outcomes have also overlooked some important nutrients such as zinc, fibre or B-complex vitamins (B_1_, B_2_, B_3_) or less-reported aspects of the diet such as alcohol and caffeine intake [10,11]. Given these limitations and the large number of newly published studies over the last few years, a comprehensive updated review on this topic is pertinent.

The purpose of this narrative literature review is to synthesize the most recent evidence, primarily from randomised controlled trials and large-scale meta-analyses, to provide an overview of what is currently known regarding macronutrient and micronutrient requirements during pregnancy, consequences of deficiencies, risks and benefits of supplementation and areas for future research. This review is non-systematic and is not intended to introduce new data or conclusions, nor does it address some aspects related to food-borne illnesses in pregnancy (listeriosis, toxoplasmosis, etc.) or food-related substances (pesticides, preservatives, heavy metals, etc.). Rather, we aimed to describe the current state of knowledge on this topic and recast it in an objective manner and in an accessible and compact form.

## 2. Macronutrients in Pregnancy

### 2.1. Energy

Energy intake is the main determinant of gestational weight gain [12]. During pregnancy, the maternal diet must provide an adequate supply of energy to support the mother’s usual requirements as well as those of the growing foetus. Extra energy is required for the synthesis of new tissue (foetus, placenta and amniotic fluid) and the growth of existing tissue (uterus, breast and maternal adipose tissue) [13]. Energy requirements during the first trimester generally do not differ from those of non-pregnant women but increase between 10 and 30 weeks of gestation when the growth of maternal and foetal tissue is greatest [13]. However, energy needs of individual women vary widely during pregnancy, depending on their physical activity levels, pre-pregnancy body mass index (BMI) and metabolic rate, hence, recommendations for energy intake should be tailored accordingly.

Global estimates suggest that energy intake during pregnancy ranges from 7710 to 9260 kJ/day, with higher intakes reported in the Americas and the Eastern Mediterranean compared with Africa, Southeast Asia and the Western Pacific [14,15]. Appropriate maternal energy intake is important to prevent poor pregnancy outcomes associated with both insufficient and excessive gestational weight gain, although there is very limited evidence from intervention trials examining the effects of energy restriction during pregnancy. A meta-analysis of three trials (*n* = 384) reported that in women who were overweight or who exhibited excessive gestational weight gain, energy restriction during pregnancy reduced maternal weight gain but had no effect on pregnancy-induced hypertension or preeclampsia (gestational hypertension with proteinuria) [16]. However, two of the three trials also reported decreases in neonatal birthweight, suggesting that energy restriction may have adverse effects on birthweight [16]. While preventing maternal obesity is important for reducing the risk of macrosomic infants, obstetric complications and birth trauma, the potential weight loss/retention benefits of energy restriction must be weighed against possible harms including foetal growth restriction. Given the absence of sufficient evidence, energy restriction is currently not advised during pregnancy and any recommendations for energy intake should be individualised based on pre-pregnancy BMI and gestational weight gain targets.

### 2.2. Protein

Protein is involved in both structural (keratin, collagen) and functional (enzymes, protein transport, hormones) biological roles. Globally, the primary sources of protein are plant-based foods such as legumes, grains and nuts (57% of daily intake) followed by animal-based foods such as meat (18%) and dairy (10%), although small amounts may also be derived from alternative sources such as algae, bacteria and fungi (mycoproteins) [17]. Protein quality is determined by its digestibility and capacity to meet the nitrogen and indispensable amino acid requirements necessary for growth, repair and maintenance. Animal protein sources are considered “complete proteins” because they provide all nine indispensable amino acids, while plant sources are “incomplete proteins” since they can be deficient in one or more indispensable proteins such as lysine or threonine [18]. Pregnant women in developed countries report consuming 14.7% to 16.1% of total energy from protein, which is adequate based on current recommendations [14]. Adjustments in protein metabolism occur within several weeks of conception in order to maintain maternal homeostasis while accommodating increased foetal demands and preparing for lactation [19]. Whole-body protein turnover studies suggest that protein turnover in early pregnancy is similar in pregnant and non-pregnant women but a 15% and 25% absolute increase in protein synthesis occurs during the second and third trimesters, respectively [19]. Concomitant decreases in maternal amino acid concentrations, urea synthesis and urinary urea excretion occur early in gestation and remain low throughout pregnancy. In well-nourished individuals, these physiological changes conserve protein and nitrogen and promote protein accretion to ensure adequate nutrient supply to the foetus [19].

Some observational studies from the UK and Spain suggest that protein intake increases birthweight independently of energy intake, maternal age, BMI or lifestyle-related variables [20,21]. However, the effect is modest, with a 1g increase in protein corresponding to a 7**–**13 g increase in birthweight. Similar findings were reported in a Cochrane review of 12 randomised trials (*n* = 6705), where an increased birthweight and a decreased risk of stillbirth and small-for-gestational-age (SGA) infants was observed following balanced energy/protein supplementation (<25% energy from protein), with no changes in gestational weight gain [22]. Isocaloric protein supplementation (i.e., where protein replaces an equal quantity of non-protein energy) had no effect on birthweight or gestational weight gain based on results from two trials (*n* = 184) [22]. Conversely, the risk of SGA increased significantly following high protein supplementation (≥25% energy from protein), with no effects on gestational weight gain or other neonatal outcomes including preterm birth, birthweight, stillbirth or neonatal death [22,23]. It should be noted that the latter findings regarding high protein supplements were derived from a single trial from 1980, which comprised 505 poor urban African American women with a history of low birthweight (LBW) infants [23]. A recent observational study of 91,637 Japanese women suggests an inverse U-shaped relationship between protein intake and foetal growth [24]. This may be due to the satiating quality of protein in the control of hunger and appetite, such that at higher levels it could have a self-limiting effect on energy intake. Overall, a protein intake of 10–25% of total energy appears to be safe, whereas the risks associated with high protein intake or supplement use cannot be ascertained from the small number of existing trials. Until more evidence is made available, protein intake during pregnancy should be kept at a moderate level (within 25% of total energy).

### 2.3. Glycaemic Index, Glycaemic Load and Fibre

Different sources of carbohydrates have variable digestion rates, thus their effects on blood glucose and insulin levels also vary. The glycaemic index (GI) quantifies glycaemic responses induced by carbohydrates from different foods [25]. High GI foods, including rice, white bread and potatoes, cause a sharp rise in blood glucose levels which decline rapidly, whereas low GI foods such as fruits or dairy have slowly digestible carbohydrates which result in a lower postprandial glucose response [25]. Glycaemic load (GL) takes into account both the quality (GI) and quantity of carbohydrates in food and is obtained by multiplying the GI by the carbohydrate content of a given food [26]. Dietary fibre describes a variety of plant-based carbohydrates that are resistant to digestion by human gastrointestinal enzymes. These include soluble fibre (fruits, vegetables, legumes), insoluble fibre (nuts, wholegrain bread or cereals) or resistant starch (cooked potato and rice) [27]. Diets high in fibre and with a low GI and GL can promote laxation, reduce blood cholesterol and modulate blood glucose and hence may be beneficial in pregnancy.

In a systematic review of two trials, low GI diets in 74 healthy women reduced the risk of large-for-gestational-age (LGA) infants [28]. In pregnancies complicated by gestational diabetes mellitus (GDM), low GI diets reduced the amount of insulin required to maintain optimal glycaemic control [28]. Dietary fibre and low GL intakes have also been associated with improved pregnancy outcomes. An observational study of 1538 women in the US showed that higher total fibre intake three months before and during early pregnancy reduced preeclampsia risk by attenuating pregnancy-associated dyslipidaemia [27]. Similarly, in 13,110 women from the Nurses’ Health Study II [29], the risk of GDM was reduced by 26% for each 10 g/day increment in total fibre in pre-pregnancy, whereas higher dietary GL correlated with a greater risk of GDM [29].

In contrast, a recent randomised trial in 139 Australian women at risk of GDM found no differences between low GI diets versus high fibre/moderate GI diets in relation to pregnancy outcomes including birthweight or incidence of GDM [30]. In a large epidemiological study of 1082 generally healthy pregnant women from a poor US city, low GI diets were associated with reduced infant birthweights and a twofold increased risk of SGA [31]. Although current evidence is limited, a low GI, low GL, and/or high fibre diet may be beneficial in preconception or early pregnancy for women at risk of developing GDM or preeclampsia or having LGA infants but it should be prescribed with caution in women at risk of SGA.

### 2.4. Fatty Acids

Essential fatty acids include linoleic (18:2 *n-6*) and alpha-linoleic acid (18:3 *n-3*) as well as their long-chain derivatives, arachidonic acid (AA), eicosapentaenoic acid (EPA) and docosahexaenoic acid (DHA) [13]. These fatty acids are key structural components of cell membranes and are vital for tissue formation. Dietary sources include oil-rich fish such as mackerel or salmon, as well as fish oil supplements (mainly omega-3) [13]. Over the course of pregnancy, maternal concentrations of essential fatty acids decrease by approximately 40%, with AA (n-6) and DHA (n-3) reducing by ~23% and ~52% by the time of birth, respectively [32]. Therefore, dietary intake of fatty acids, particularly long-chain polyunsaturated fatty acids (PUFAs) such as DHA and EPA, is important during pregnancy to meet the requirements of the mother as well as the developing foetus. DHA can influence the development of the brain and retina in the foetus, while EPA may reduce the synthesis of thromboxane A2 from AA, thereby potentially reducing the risk of preeclampsia and timing to parturition [33].

Benefits of supplementation with long-chain *n-3* PUFAs during pregnancy have attracted substantial interest in recent years; however, evidence remains inconsistent. A 2006 Cochrane meta-analysis (six trials, *n* = 2783) found that the use of marine oil supplements or other prostaglandin precursors did not prevent gestational hypertension, preeclampsia or eclampsia, while the effects on birthweight and duration of gestation were inconsistent [34]. A recent update of this review (70 trials, *n* = 19,927) reported that omega-3 supplementation during pregnancy reduced preterm birth (<37 weeks’ gestation) and early preterm birth (<34 weeks’ gestation) but also contributed to prolonged gestation (>42 weeks’ gestation) [33]. Some evidence of variable quality indicated a reduction in LBW, perinatal death and admission to a neonatal intensive care unit (NICU) in women receiving omega-3 supplements but a small increase in LGA was also observed [33]. In other meta-analyses, omega-3 supplements had no effects on recurrent preterm birth or recurrent intrauterine growth restriction [35,36]. Supplementation with PUFAs may be beneficial for preventing preterm birth and improving neonatal outcomes, particularly in high-risk pregnancies but they can also increase the incidence of post-term pregnancies and LGA. An additional 23 studies are currently ongoing, some of which include >5000 women [33] and evaluation of these studies is needed before PUFA supplements can be recommended for use in pregnancy.

## 3. Micronutrients in Pregnancy

### 3.1. Folate

Folate is a water-soluble B vitamin present in leafy green vegetables, yeast extract and citrus fruits such as oranges. Some breads and breakfast cereals are fortified with folic acid- the synthetic and more stable form of folate [8]. Folate functions as a coenzyme in one-carbon transfers during methylation cycles and is therefore integral for the synthesis of DNA and neurotransmitters. It is also involved in amino acid metabolism, protein synthesis and cell multiplication, making it particularly important during embryonic and foetal stages of pregnancy where there is rapid cell division and tissue growth [8]. Folate deficiency results in the accumulation of homocysteine, which can increase the risk of adverse outcomes including preeclampsia and foetal anomalies. Women in areas where malaria and/or sickle cell disease are prevalent are at increased risk of folate inadequacy [8]. Serum folate reflects recent intake and levels below 10 nmol/L suggest deficiency, while red blood cell folate indicates folate storage and is considered deficient at levels below 340 nmol/L [37]. There is no universal cut-off for deficiency in pregnancy but folate concentrations usually decline during gestation, likely due to increased folate demands to facilitate increases in blood volume, hormonal changes and foetal and uteroplacental organ development [37].

Supplementation with folic acid during preconception and early pregnancy is critical and can prevent 40–80% of neural tube defects such as spina bifida [38]. Because the neural tube develops in the first four weeks of pregnancy, the protective effects of folic acid supplements are diminished after pregnancy is established [13]. In addition to consuming a folate-rich diet, an intake of 400 μg/day of folic acid from fortified foods, supplements or both (total intake ~600 μg/day) is recommended for all reproductive age women from at least one month before conception until at least 12 weeks’ gestation [37,39]. Higher doses (4**–**5 mg/day) are recommended for women at high-risk (history of neural tube defects, diabetes and anticonvulsant medication use) while intermittent doses (5 mg/week) may be used for women with poor compliance to medication, side-effects or poor lifestyle habits including inadequate diets, smoking or alcohol consumption [39].

Evidence pertaining to the use of folic acid for preventing other pregnancy complications is less clear. The most recent evidence comes from a 2015 Cochrane review (five trials, *n* = 7391), which confirmed the protective effects of folic acid supplements in relation to neural tube defects but found no effects on miscarriage or other birth defects including congenital heart defects, cleft lip or cleft palate [8]. There was insufficient evidence to assess the effects of folic acid intake/supplementation on occurrence or recurrence of other birth defects or on maternal outcomes, an area for future research [8]. An earlier Cochrane review in 2013 (31 trials, *n* = 17,771) found that folic acid improved pre-delivery serum folate and megaloblastic anaemia but had no effect on preterm birth, stillbirth/neonatal death, birthweight, pre-delivery haemoglobin or red cell folate [6]. It should be noted that folic acid supplementation can mask vitamin B_12_ deficiency and can contribute to possible unwanted side effects, including twin/multiple pregnancies which carry a higher risk of perinatal complications. Nevertheless, on the balance of benefit and risk, adequate folate intake through supplements and diet is advised for all women during preconception and pregnancy.

### 3.2. Vitamin A

Vitamin A is a fat-soluble vitamin derived from either preformed retinoids or provitamin carotenoids. Retinoids, such as retinal and retinoic acid, are obtained from animal sources including eggs, dairy, liver and fish liver oil. Carotenoids such as beta-carotene are obtained from plant sources such as dark or yellow vegetables including kale, sweet potatoes and carrots and can be converted to vitamin A in the liver where vitamin A is stored [40]. Physiological functions of vitamin A include vision, growth, bone metabolism, immune function and gene transcription as well as antioxidant activities. Some additional vitamin A is needed during pregnancy to support growth and tissue maintenance in the foetus and to provide foetal reserves and aid in maternal metabolism [40]. Pregnant women have a basal requirement of 370 µg/day and a daily intake of 770 µg/day is recommended, which is thought to be supplied by maternal liver stores in women without deficiency [41].

Hypovitaminosis A is determined by either a history of night blindness or serum/plasma retinol concentrations below 0.7 μmol/L (subclinical vitamin A deficiency). Night blindness affects up to 7.8% of pregnant women globally (9.8 million), while 15.3% (19.1 million) are considered deficient based on serum retinol concentrations [42]. Studies suggest that maternal night blindness correlates with a higher risk of infant mortality and LBW infants [42], yet vitamin A supplements have not shown any benefit in preventing these outcomes. A Cochrane meta-analysis of 19 trials and **>**310,000 women reported that vitamin A supplementation during pregnancy had no effects on maternal or new-born death, stillbirth, LBW, preterm birth or anaemia in the new-born but reduced the risk of maternal anaemia, infection and night blindness, particularly in vitamin A-deficient women [40]. Effects of vitamin A supplements are likely to vary by baseline deficiency status and further studies are needed to explore this variation, as well as the optimal dose and duration required and the potential benefits of vitamin A for preventing infection. Because retinol is associated with teratogenic effects, an upper limit of 10,000 IU per day (3000 µg retinol) has been established and the non-toxic form (beta-carotene) is preferred during pregnancy. Taken together, current evidence does not support the use of vitamin A supplementation for improving pregnancy outcomes, especially in developed countries where deficiency is rare. In deficient women, supplementation may be initiated after careful assessment of current intake and with regular monitoring to prevent toxicity.

### 3.3. Vitamin B_1_ (Thiamine), Vitamin B_2_ (Riboflavin), Vitamin B_3_ (Niacin), Vitamin B_6_ (Pyridoxine) and Vitamin B_12_ (Cyanocobalamin)

B-complex vitamins including vitamins B_1_ (thiamine), B_2_ (riboflavin), B_3_ (niacin), B_6_ (pyridoxine) and B_12_ (cyanocobalamin) are water-soluble vitamins required for the production and release of energy in cells and for the metabolism of protein, fat and carbohydrates. These vitamins act as coenzymes in several intermediary metabolic pathways for energy generation and blood cell formation [43]. Vitamin B_12_ functions alongside folate to convert homocysteine to methionine, a process which is essential for the methylation of DNA, RNA, proteins, neurotransmitters and phospholipids [44]. Deficiency in these vitamins can therefore impact on cellular growth as well as on nerve tissue development due to its high-energy demand. Most prenatal supplements include B-complex vitamins, yet, with the exception of vitamin B_12_, the individual metabolic roles of B-vitamins are not well defined in pregnancy.

B-complex vitamins are obtained primarily from animal sources including meat, poultry, fish and dairy products and can also be found in fortified cereals, legumes and leafy green vegetables [13]. The requirement for these vitamins is heightened in pregnancy due to increased energy and protein needs, particularly during the third trimester. However, adaptive responses during pregnancy reduce urinary excretion of some B-vitamins, including riboflavin, to help meet increasing demands [13]. Vitamin B_12_ insufficiency is proposed to affect 25% of pregnancies worldwide, while global estimates of deficiencies in other B-complex vitamins are unavailable [3,44].

Some studies suggest that thiamine deficiency may impair foetal brain development due to subclinical metabolic disturbances in the thiamine-dependant enzyme systems involved in lipid and nucleotide synthesis in the brain [45,46]. Deficiencies in riboflavin and niacin have been correlated with preeclampsia, congenital heart defects and LBW infants; however, evidence regarding the benefits of supplementation in preventing these outcomes is sparse [47,48,49]. Similarly, increased periconceptional intakes of thiamine, niacin and pyridoxine have been correlated with decreased nausea and reduced risk of orofacial cleft but supplementation trials are yet to establish causality [3]. A systematic review of four trials (*n* = 1646) examining pyroxidine supplementation found that it did not prevent hypertensive disorders of pregnancy and inadequate data were available to examine its effects on other maternal or neonatal outcomes [50]. Low vitamin B_12_ levels can lead to increased homocysteine concentrations, with subsequent adverse outcomes including placental abruption, stillbirths, LBW and preterm delivery [51]. A meta-analysis of 18 longitudinal studies including individual patient data (11,216 observations) reported that B_12_ concentrations <148 pmol/L were associated with an increased risk of LBW infants and preterm birth [51]. Like folate, B_12_ deficiency has also been linked with increased risk of neural tube defects including spina bifida [51]. Nevertheless, there is insufficient evidence to confirm the potential benefits of B-vitamin supplements during pregnancy and, given the potential risks associated with deficiencies, further research by means of randomised trials is highly warranted.

### 3.4. Vitamin C and E

Vitamin C (ascorbic acid) is an essential water-soluble vitamin, whereas vitamin E represents eight fat-soluble, plant-derived compounds: four tocopherols and four tocotrienols (alpha, beta, gamma, delta), with naturally sourced alpha-tocopherol as the most biologically active form [52,53]. Many fruits and vegetables including guava, citrus fruits, tomatoes and broccoli are rich in vitamin C, while vitamin E is found in nuts, wheatgerm oil, vegetable oils and some leafy green vegetables [52,53]. Both vitamins C and E function synergistically to promote antioxidant defences and inhibit free radical formation to prevent oxidative stress. Hence, they are often supplemented concurrently to utilise this relationship [52]. Vitamin C is also involved in synthesising collagen, a primary component of connective tissue and has an important role in mobilising iron from stores and enhancing the absorption of dietary iron, so it can aid in preventing megaloblastic and iron-deficiency anaemia [13,53]. Vitamin C is actively transported across the placenta which leads to reduced maternal plasma levels and increased requirements from 30–70 mg/day in non-pregnant adults to 60–85 mg/day during pregnancy and lactation [53]. Conversely, placental transfer of vitamin E is less efficient and losses of vitamin E to the foetus are thought to be minimal. An intake of 7–10 mg/day of alpha-tocopherol is therefore recommended for adults irrespective of pregnancy status [54]. Unless otherwise indicated, a healthy varied diet is likely sufficient for meeting intake requirements for both vitamins.

Oxidative stress is thought to be a key mechanism underlying the pathophysiology of several pregnancy complications including preeclampsia, preterm birth, intrauterine growth restriction (IUGR) and premature rupture of membranes (PROM) [54,55,56]. This has prompted interest into whether antioxidants such as vitamins C and E may be protective against these conditions. Two Cochrane reviews examining vitamin C (*n* = 29 trials, *n* = 24,300) and vitamin E (21 trials, *n* = 22,129), alone or in combination with other supplements, both reported a reduced risk of placental abruption but an increase in self-reported abdominal pain [52,53]. Gestational age at birth was increased slightly with vitamin C and, when provided alone, vitamin C decreased the risk of PROM at both preterm and at term. In contrast, vitamin E taken alone or in combination with vitamin C increased the risk of term PROM [52,53]. No other effects were reported for either vitamin. Based on current evidence, supplementation with vitamins C or E is not recommended during pregnancy and their roles and interactions in relation to PROM require further study.

### 3.5. Vitamin D

Vitamin D is a fat-soluble hormone known for its role in maintaining calcium homeostasis and bone integrity. Extraskeletal functions of vitamin D are also widely recognised including its role in glucose metabolism, angiogenesis, inflammation and immune function, as well as in regulating gene transcription and expression [57]. Vitamin D is obtained primarily via subcutaneous synthesis following ultraviolet-B radiation (sun exposure) and is also found in a few foods including oily fish or fortified dairy products and in supplements in the form of cholecalciferol (vitamin D_3_) or ergocalciferol (vitamin D_2_) [58]. Following ingestion or synthesis, vitamin D is first hydroxylated in the liver to form 25-hydroxyvitamin D (25(OH)D), the major circulating form and most common measure of vitamin D status and then in the kidney to form 1,25-dihydroxyvitamin D (1,25(OH)_2_D_3_), the biologically active form [59]. Serum 25(OH)D levels <75, <50 and <25 nmol/L are used to define insufficiency, deficiency and severe deficiency, respectively. Globally, it is estimated that 40–98% of pregnant women are vitamin D-deficient, while 15–84% are severely deficient [60]. Deficiency can be attributed to low intake of fortified foods, highly pigmented skin and lack of sunlight exposure due to sedentary indoor lifestyles and use of sunscreen and/or protective clothing to prevent skin cancer [57]. While it is important to control vitamin D status and to prevent deficiency across both pregnant and non-pregnant populations, there is currently no consensus on the optimal levels of 25(OH)D required to improve extraskeletal health or pregnancy outcomes.

In pregnancy, the foetus relies completely on maternal vitamin D stores for its development [58]. Levels of 1,25(OH)_2_D_3_ increase from early pregnancy and continue to rise 2-3 fold until delivery, reaching levels >700 pmol/L which would be toxic in non-pregnant individuals [7]. This process is unique to pregnancy and is dependent on the bioavailability of the 25(OH)D substrate but independent of calcium metabolism [7]. Maternal vitamin D deficiency has been associated with neonatal rickets [61] as well as multiple adverse pregnancy outcomes including GDM, preeclampsia [62,63], preterm birth [64] and SGA infants [65]. In a Cochrane meta-analysis of 15 trials including 2833 women, vitamin D supplements during pregnancy reduced the risk of preeclampsia, LBW and preterm birth; however, when combined with calcium, the risk of preterm birth was increased [58]. A more recent systematic review of observational and intervention studies concluded that maternal 25(OH)D status is modestly associated with offspring birthweight, bone mass and serum calcium concentrations; however, conclusions regarding other outcomes could not be reached with the available evidence [66]. Overall, preventing and treating vitamin D deficiency in pregnancy is important for optimising maternal and foetal bone health and for supporting foetal growth but there is limited evidence supporting the use of vitamin D supplements for improving other pregnancy outcomes.

### 3.6. Calcium

Calcium is an essential nutrient for bone mineralisation and a key intracellular component for maintaining cell membranes. It is involved in several biological process including signal transduction, muscle contraction, enzyme and hormone homeostasis, as well as neurotransmitter release and nerve cell function [67]. Milk and dairy products are the best sources of calcium and it can also be derived from leafy green vegetables, nuts or fortified foods including flour and dairy alternatives (e.g., soya products) [13]. In pregnancy, calcium is actively transported across the placenta and maternal calcium demands increase, particularly during the third trimester. More efficient uptake and utilisation of calcium occurs naturally during pregnancy via physiological adaptations, including increased calcium absorption stimulated by hormones (vitamin D, oestrogen, lactogen and prolactin) and enhanced retention of calcium by kidney tubules [13]. Increased calcium needs may therefore be met by diet alone (1.2 g/day recommended); however, supplementation of 0.3–2.0 g/day is recommended by some to preserve maternal calcium balance and bone density and to support foetal development, particularly in women with low dietary calcium intake (<1 g/day) [67].

Low maternal calcium intake can contribute to osteopenia, paraesthesia, muscle cramps, tetanus and tremor in the mother, as well as delayed growth, LBW and poor foetal mineralisation in the foetus, although evidence for the latter has been inconclusive [68,69]. Recent evidence suggests that women with low calcium intake are also at higher risk of developing hypertensive disorders of pregnancy. A 2013 report by the World Health Organization (WHO) [69] combined data from two Cochrane reviews [67,68] totalling 21 trials and over 90,000 women. Results showed that calcium supplementation reduced the risk of preeclampsia by more than 50% in all women, irrespective of their baseline calcium intake or risk profiles for hypertension. Protective effects were more prominent in women with lower baseline dietary calcium intakes (<900 mg/day) and in women at higher risk of preeclampsia [69]. A 2018 update of one Cochrane review showed that women receiving <1 g/day had reductions in high blood pressure and NICU admissions, while those receiving >1 g/day had modest reductions in preterm birth but increases in HELLP syndrome (haemolysis, elevated liver enzymes and low platelets) [68]. However, the quality of evidence was low for outcomes other than hypertensive disorders and results should be interpreted with caution [68]. Currently, the WHO recommends supplementation with 1.5–2.0 g/day of calcium during pregnancy for women at high risk and/or women with low dietary calcium intake [69].

### 3.7. Iodine

Iodine is an essential nutrient for regulating growth, development and metabolism via the biosynthesis of thyroid hormones including thyroxine (T4) and triiodothyronine (T3). Iodine is obtained mainly from fortified salt but it can also be sourced from kelp and seafood, as well as dairy or plant foods in places with iodine-fortified animal feed or iodine-rich soil [70]. During pregnancy, metabolic demands and hormonal alterations result in a substantial increase in iodine requirements. This is because early in gestation, thyroid hormone production increases by 50% and renal excretion of iodine increases by 30–50%, while later in gestation, iodine passes the placenta for foetal thyroid hormone production [71]. Maternal and foetal thyroid hormones regulate key processes in the development of the foetal brain and nervous system, including the growth of nerve cells, the formation of synapses and myelination [72]. Only small amounts of iodine (150–290 µg/day) are required to prevent deficiency, yet iodine deficiency disorders (IDDs) remain the most common cause of preventable brain and cognitive impairments worldwide. Although global estimates in pregnant women are not available, approximately 1.8 billion people worldwide have iodine insufficiency, with Europe and Southeast Asia having the highest proportion (44%) and number (540 million) of individuals with iodine insufficiency, respectively [70]. IDDs in the foetus range from mild intellectual impairments to more severe and irreversible neurologic and physical stunting (endemic cretinism or congenital hypothyroidism). Other consequences of IDDs in pregnancy include maternal and foetal goitre, lower intelligence quotient (IQ) scores in the offspring, increased pregnancy loss and infant mortality [70].

There is limited evidence to date regarding the benefits and harms of iodine supplementation before, during or after pregnancy. A Cochrane review of 11 trials and >2700 women found that in settings of low to moderate iodine deficiency, women receiving iodine supplements during pregnancy had a greater likelihood of experiencing digestive intolerance but a lower likelihood of experiencing adverse effects associated with postpartum hyperthyroidism [70]. There were no differences in preterm birth, LBW, neonatal hypothyroidism or maternal hyper- or hypothyroidism during pregnancy or postpartum. In settings with severe iodine deficiency, a 34% reduction in perinatal mortality was observed in women receiving iodine supplements; however, this result was not statistically significant and was derived from a single study [70]. Despite a lack of high-quality evidence supporting the use of iodine supplementation in pregnancy, the importance of adequate maternal iodine status for foetal development is well-established. Hence, a dietary iodine intake of 250 µg/day is recommended for pregnant and lactating women [73]. In regions where only 20–90% of households use iodized salt, periconceptional supplementation (150–250 µg/day) may be required until universal salt iodization programs are implemented and/or upscaled [73].

### 3.8. Iron

Iron is a vital nutrient and cofactor for the synthesis of haemoglobin and myoglobin, as well as for several cellular functions including oxygen transport, respiration, growth, gene regulation and the proper functioning of iron-dependent enzymes [74,75]. Intracellular homeostasis and proper balance of iron stores are therefore tightly regulated [74]. Yet, iron deficiency remains the leading single-nutrient deficiency worldwide, affecting over two billion people including ≥30% of pregnant women in the industrialized world, with higher rates in developing countries [76]. Iron deficiency results from poor dietary intake of absorbable iron (or insufficient intake to meet increased demands in pregnancy) and/or loss of iron through parasitic infections (e.g., hookworm) or blood loss [77]. Plant-based foods such as green leafy vegetables contain non-heam iron, which makes up the major fraction of dietary iron. However, haem-iron from foods such as animal meats and fish has a higher bioavailability and is absorbed more efficiently, making it the main source of dietary iron for mammals [74,75].

In pregnancy, the demand for iron increases from 0.8 to up to 7.5 mg/day of absorbed ferritin, although the exact upper limits in the third trimester are debated [76]. This increased demand is needed to expand maternal erythrocyte mass, fulfil foetal iron needs and compensate for iron losses (e.g., blood loss at delivery) [75]. Maternal iron requirements therefore exceed average absorbable iron intakes and, in turn, the risk of developing iron deficiency anaemia is increased in pregnancy [76]. An estimated 38.2% of pregnant women globally are anaemic (defined by WHO as haemoglobin <110 g/L with a recognised 5 g/L decrease in the second trimester) [78] and the prevalence is particularly high in Southeast Asia (80%), as well as the Eastern Mediterranean (65%) and Africa (47%) [76].

Iron deficiency and/or anaemia have been associated with greater risk of preterm birth, LBW or SGA infants, impaired maternal function and decreased defences against infection, as well as abnormal psychomotor development and cognitive function in infancy [74,77]. However, evidence from iron supplementation trials has been inconclusive. A 2015 Cochrane meta-analysis [77] of 44 trials (*n* = 43,274) reported a 70% and 57% reduction in maternal anaemia and iron deficiency at term, respectively, in women receiving preventive iron supplements compared with no iron or placebo. There were no differences in neonatal or maternal mortality or congenital anomalies and although women taking iron supplements had lower absolute numbers of LBW and preterm infants, these differences were not statistically significant [77]. Based on the available evidence, iron supplementation can prevent maternal deficiency and anaemia but its benefits for other maternal and neonatal complications is less clear. Periconceptional supplementation with 30 to 60 mg/day of elemental iron is recommended for women during pregnancy for preventing anaemia, with 60 mg as the preferable dose in regions where maternal anaemia affects >40% of pregnancies [79].

### 3.9. Zinc

Zinc is an important catalytic component of over 200 enzymes and structural component of several nucleotides, proteins and hormones. It has critical ubiquitous roles in biochemical functions including protein synthesis and nucleic acid metabolism, as well as cellular division, gene expression, antioxidant defences, wound healing, vision and neurological and immune function [80]. Zinc is present in many foods but higher levels can be found in meat, seafood, milk and nuts, whereas diets high in fibre or phytate can reduce the bioavailability of zinc [80]. Zinc status can be assessed by measuring plasma or serum zinc levels, zinc-dependent enzyme levels or 24-h urinary zinc excretion, although values can vary by age, sex, time of day and physiological factors such as stress or infection [80]. This makes it difficult to establish cut-offs and accurately assess the prevalence of zinc deficiency. Nevertheless, it is estimated that 82% of pregnant women have inadequate zinc intakes and that pregnant and lactating women consume ~9.6 mg/day of zinc, well below the recommended intake of 15 mg/day during the second and third trimesters [81,82].

Zinc deficiency is proposed to contribute to about half a million maternal and child deaths annually, mostly in developing countries [83]. Zinc deficiency in pregnancy has been associated with impaired immunity, prolonged labour, preterm and post-term births, intrauterine growth retardation, LBW and pregnancy-induced hypertension [79,84]. Although rare, severe zinc deficiency including that resulting from inherited defects in zinc absorption (acrodermatitis enteropathica) can lead to congenital malformations and pregnancy loss [84]. Two separate meta-analyses [84,85] reported that zinc supplementation during pregnancy reduced the incidence of preterm birth by 14% but had no effects on birthweight, hypertensive disorders or neonatal mortality [84,85]. The reduction in preterm birth was seen mainly in women from low-income settings and hence may reflect improvements in the poor baseline nutritional status of these women, rather than isolated effects of zinc supplements per se. It is also plausible that zinc may reduce preterm birth via reducing maternal infection, a primary cause of prematurity [84]. To date, benefits of zinc nutriture during pregnancy have not been demonstrated and as zinc deficiency is likely a reflection of poor diet, strategies to improve overall maternal nutrition are likely to yield more tangible health benefits than the use of zinc supplements alone.

### 3.10. Alcohol and Caffeine

Global estimates suggest that approximately 10% of women consume alcohol during pregnancy, with the prevalence highest in European regions and lowest in Eastern Mediterranean and Southeast Asian regions [86]. Alcohol abuse or heavy consumption in pregnancy (defined as >80 g or ≥8 standard drinks [SD]/day [87]) is known to be associated with foetal alcohol spectrum disorders (FASD). FASD are a cluster of congenital defects, growth restriction, distinctive facial appearance, and/or neurodevelopmental problems including mental retardation [13,88]. Risks of first trimester miscarriage, preterm birth and SGA infants are also increased in women with high or regular alcohol intakes (≥4 SD/day or ≥8 SD/week) [87,89].

Implications of occasional or low to moderate alcohol consumption (<1 SD/day or 0**–**6 SD/week [87]) on maternal and infant health are less clear. In two systematic reviews of 9**–**22 observational studies, prenatal alcohol exposure of any amount was associated with poorer cognition, behaviour and mental development in the offspring [90,91]. Conversely, two other systematic reviews [88,92], one of which included 46 studies and >100,000 women [88], concluded that there was no convincing evidence of adverse effects from low to moderate prenatal alcohol exposure. There were substantial inconsistencies and limitations in existing studies, including lack of adjustment for sociodemographic confounders and potential reporting bias leading to over or underestimation of drinking patterns. As such, definitive conclusions regarding safe drinking limits in pregnancy cannot be drawn from current evidence and abstinence from alcohol intake during pregnancy is advised.

Caffeine is a trimethylxanthine alkaloid and the most commonly used psychoactive substance worldwide. Coffee is the most common source of caffeine but other foods and beverages including chocolate or cocoa, tea, cola and some medications also contain caffeine [93]. Paraxanthine, the primary metabolite of caffeine, crosses the placenta and antagonises receptors for adenosine (A1), an endogenous modulator of neuronal excitability. This in turn promotes stimulatory activities in maternal and foetal whole brain, with potential effects on the metabolic activity of both mother and foetus [93]. During pregnancy, maternal clearance of caffeine slows down substantially and its half-life triples in the second and third trimesters, while the foetus has inadequate amounts of the enzyme needed to metabolise caffeine [93]. Excess intakes of caffeine can promote vasoconstriction in uterine and placental circulations and increase foetal heart rate and arrhythmias, with potentially harmful effects on foetal growth and development [94].

While it is biologically plausible that caffeine may adversely affect pregnancy outcomes, studies of its effects in pregnancy have been inconsistent. Several meta-analyses of observational studies (some including >90,000 or >130,000 women) reported that caffeine intake during pregnancy was associated with LBW and pregnancy loss in a dose-response manner, whereby each 100-150 mg/day increment in caffeine intake was associated with a 3–13% increased risk of LBW and a 7–19% increased risk of pregnancy loss [9,95,96,97]. However, a Cochrane meta-analysis of two trials compared caffeinated coffee (*n* = 568) with decaffeinated coffee (*n* = 629) and found that reducing caffeine intake by an average of 182 mg/day had no effects on birthweight, preterm birth or SGA infants [93]. The lack of consistent evidence precludes conclusions regarding any harms or benefits of caffeine intake in pregnancy; however, pregnant women with high intakes (>300 mg/day) are advised to reduce their caffeine intake until safe upper limits are established.

## 4. Discussion of Findings and Limitations

### 4.1. Summary of Evidence

Adequate energy and nutrient intake is pivotal in pregnancy and should begin before conception and continue throughout pregnancy to support usual maternal needs while laying down the stores required for foetal development and for lactation [13]. Requirements for several nutrients increase during pregnancy to meet maternal and foetal demands, which often exceed those met by physiological adaptations, necessitating an increased intake to meet these demands. Balanced energy and protein intake is recommended, taking into account pre-pregnancy BMI and individualised targets for gestational weight gain. Iron and folic acid supplements, in addition to a folate- and iron-rich diet, are important for preventing anaemia and neural tube defects, respectively. Vitamin D and calcium supplements are also recommended during pregnancy to ensure optimal maternal stores and to support foetal growth and bone development, as well as to prevent hypertensive disorders, particularly in women at high risk of deficiency or insufficient dietary intake. Conversely, intakes of vitamin A should be limited to prevent teratogenicity, while alcohol and excess caffeine should be avoided to minimize associated risks to the foetus. Other nutrients including PUFAs, zinc, vitamins C and E and B-complex vitamins have critical roles in maternal and foetal health and should be acquired through a balanced diet. However, further research is needed before their use in pregnancy in the form of supplements can be supported. Table 1 presents a summary of the evidence discussed in this review including recommendations for interventions and daily intakes based on WHO and Institute of Medicine guidelines, respectively.

### 4.2. Limitations and Future Directions

Evidence regarding the importance of macro- and micro-nutriture in pregnancy is rapidly and continually evolving. Yet, there remain important knowledge gaps in our understanding of nutrient interactions, optimal timing and dosages required during pregnancy and the effects of specific nutrients on immediate, short-term and long-term outcomes. Mechanisms and outcomes related to micronutrient function in human pregnancy are typically inferred from in vitro studies and experimental animal models, as well as human observational studies and a limited number of trials. However, current knowledge regarding specific causal mechanisms is limited and further studies should aim to elucidate how micronutrients function in vivo through the materno–placental foetal axis to influence pregnancy outcomes. Epigenetic mechanisms and genetic variants can also interact with dietary intakes to influence pregnancy outcomes but these have seldom been explored in pregnancy. For instance, biomarkers such as microRNAs (miRNAs), which regulate the expression of up to 30% of the human genome, can reveal important epigenetic mechanisms which can influence developmental processes and subsequent pregnancy outcomes [98]. For some nutrients such as vitamin D, single nucleotide polymorphisms (SNPs) in receptors or metabolising enzymes are proposed to influence individual responses to and benefits from, supplementation [99,100]. Further studies exploring miRNAs, SNPs and other genetic factors are needed to clarify the impact of nutrition on the epigenome during foetal development and to develop personalised nutrition approaches (e.g., nutrigenomics) in future.

Neonatal development is influenced by maternal nutritional status in both the preconception period and throughout pregnancy [20]. Yet, clinical examinations and nutrient intakes are often not assessed in the preconception and periconceptional phases, when placentation and embryogenesis would likely benefit most [3]. In fact, for many micronutrient studies, data collection is performed at a single timepoint during pregnancy. Such studies often fail to identify the “critical windows” of opportunity whereby measuring and treating certain deficiencies can promote the greatest benefit. Single timepoint measures also give rise to flawed assessments because micronutrient concentrations (which are often used as biomarkers to estimate status and physiological need) can change over the course of pregnancy due to plasma volume expansion and other adaptations of the pregnant state [3]. Future studies aiming to relate maternal dietary intake to new-born health parameters should account for these physiological changes and should perform data collection in the preconception period, longitudinally across various timepoints during the pregnancy, and, if relevant, during lactation.

Another limitation is that a large proportion of nutrition research is derived from observational and population-based studies. These studies offer important insights for public health as well as much needed information on the prevalence of nutritional deficiencies and their potential implications. However, observational studies, particularly retrospective designs, can be heavily influenced by confounding and often rely on self-reported dietary intakes which can be subject to reporting and recall bias. Socioeconomic status (SES) and lifestyle-related variables, if unaccounted for, can also introduce confounding bias to these studies. For example, low SES or financial stress can affect pregnancy outcomes directly (e.g., via increased stress responses, inflammation and susceptibility to infection) or indirectly (e.g., via decreased timely access to health care) [101]. Similarly, poor health literacy (i.e., lack of knowledge around risk factors or non-adherence to medications or prenatal vitamins) or lifestyle habits such as smoking have been shown to increase the risk of adverse pregnancy outcomes including stillbirth and LGA infants [102,103]. Failing to account for these factors in observational studies can lead to false conclusions regarding the impact of maternal nutrition on pregnancy outcomes, particularly in certain settings such as low SES or minority communities where access to healthcare and resources is problematic. Observational studies also need to consider confounding variables related to the specific nutrient or disease under investigation. For example, studies examining vitamin D in pregnancy should account for variables such as skin pigmentation or changes in sun exposure to assess whether these factors influenced vitamin D levels over a given study period. Similarly, studies examining GDM may overlook unique confounders such as previous history of GDM or family history of type 2 diabetes, which can influence GDM risk profiles of the study population, thereby distorting the relationship between a treatment, risk factor or exposure with this specific clinical outcome.

For certain substances such as alcohol, levels of consumption may be underreported due to social desirability bias and fear of stigma, leading to invalid exposure data and erroneous results. In other words, studies may report an association between low or moderate alcohol intake and adverse outcomes, whereas the level of consumption was, in reality, much higher than reported [104]. Underreporting is likely to be a factor in most alcohol studies; however, better data may be derived from areas where some alcohol intake during pregnancy is socially acceptable such as in some European countries [104,105]. Other outcomes, such as neurocognitive or socioemotional development of offspring, can be challenging to assess due to a lack of standardized and validated methods which enable these outcomes to be measured comparably across cultures [3].

Imprecise exposure measures, residual confounding and collinearity among dietary exposures are key drawbacks of observational studies, often leading to overinflated or underestimated results, which may explain some of the inconsistent findings reported in observational studies compared with intervention trials. On the other hand, results may diverge simply because these studies examine different exposures (i.e., trials include point-exposure in which exposure is applied and fixed at baseline, usually after the first trimester, whereas observational studies can capture nutritional status and capacity over longer periods of time). In any case, the likelihood and magnitude of biases in observational studies is difficult to quantify and generalise for maternal nutrition research as a whole, as these tend to vary by study design, participants and the particular nutrient or disease in question. The post-hoc nature of many observational studies also means that assessment of confounding variables is not always possible, since data collection has ceased. Moreover, adequate adjustment for confounders relies on the assumption that the tools used are able to capture what they are intended to and also rely on accurate reporting by participants [104]. These inherent complexities make it difficult to ascertain the extent and direction of biases encountered in observational studies of maternal nutrition. Nevertheless, future research should aim to identify the unique and often context-specific confounders and to control and/or acknowledge the portion of the exposure-outcome association that is driven by factors other than the exposure under study.

Randomised controlled trials can, to some extent, address the issues posed by observational studies. However, for several micronutrients such as zinc, vitamins C and E and B-complex vitamins, randomised trials in pregnancy remain sparse to absent, while other micronutrients such as vitamin D have attracted substantial interest but lack trials of sufficient size to explore effects on outcomes such as preeclampsia or preterm birth. Similarly, trial data examining the effects of in utero macronutrient and micronutrient exposure on long-term outcomes of offspring are lacking and greatly needed. Although considered the gold-standard in many respects, randomised trial designs can be difficult to use for assessing non-linear or dose-response effects. To some extent, this makes them ill-suited for nutrition research, particularly given the polyvalent nature and non-monotonic responses of nutrients, as well as the long latency periods required to observe certain effects and/or outcomes [106]. Existing nutrition trials are also limited by a number of factors, including poor compliance, short durations, heterogeneous populations and dietary assessment methodologies (24-hour recall, food records, food frequency questionnaires etc.), as well as small sample sizes and lack of statistical power. This is further complicated by a lack of defined knowledge around blood concentrations which constitute deficiency for particular nutrients, optimal target concentrations required for these nutrients and the ideal dosage, timing and duration of interventions needed to achieve the desired outcomes.

Most intervention studies commence supplementation during or after the first trimester, hence, the confirmatory effect of intervening earlier remains an unmet research need. This is particularly relevant in undernourished populations where certain micronutrients may be required before and during conception to promote normal placentation and embryogenesis and realise the full effect of the nutrient. Many intervention trials have also used co-supplementation regimens, such as trials of vitamin D and calcium or have not excluded women taking prenatal supplements. This can mask effects and make it difficult to delineate the potential benefits of one supplement over another. As mentioned earlier, individual social and economic circumstances which influence pregnancy outcomes (such as educational level and health literacy, as well as socioeconomic status and access to healthcare) are often neglected in nutrition trials and warrant further consideration. The quality of food sources is another important consideration and should be assessed in studies measuring dietary intakes, for instance by using protein quality scores or diet quality indices or evaluating the quality of fats or oils used for cooking. It is also unclear whether specific subgroups of women may benefit more than others from a particular nutritional intervention and whether certain thresholds exist whereby an intervention ceases to incur additional benefits or in fact has detrimental effects, as is the case for iodine or vitamin A for example. Finally, it should be noted that clinical guidelines often rely on combined effect estimates derived from these randomised trials in the form of synthesized meta-analyses, many of which are summarized in the present review. These meta-analyses extend the highest level of evidence and offer important insights into the efficacy of nutritional interventions on pregnancy outcomes. However, they are no panacea against poorly conducted individual studies which, when included in meta-analyses, can often compromise the quality and certainty of the overall evidence.

## 5. Conclusions

In summary, the importance of maintaining a healthy and varied diet before and during pregnancy should not be underestimated. Nutritional deficiencies during pregnancy remain a public health concern, particularly in underprivileged and high-risk populations but the variable quality of current evidence makes the full extent of their burden difficult to quantify. Further, large-scale and robustly designed studies during preconception and throughout pregnancy are needed to assess the full consequences of macronutrient and micronutrient deficiencies on pregnancy outcomes and to clarify the potential impact of nutritional interventions in improving these outcomes.

## Figures and Tables

**Table 1:** Summary of evidence regarding macronutrient and micronutrient intakes during pregnancy.

Nutrient	Recommendations for Interventions/Supplement Use ^1^	Non Pregnant Adult Females (19–50 years) ^2^	Pregnant Adult Females (19–50 years) ^2^
**Macronutrients**			
Energy	Energy restriction reduces GWG but could adversely affect birthweight and is currently not recommended in pregnancy	EER (kcal/day) ^3^ = 354 − (6.91 × age [year]) + PA × [(9.36 × weight [kg]) + (726 × height [m])]	Non pregnant EER + 340 and 452 kcal/day in 2nd and 3rd trimesters
Protein	Balanced energy/protein supplements (≤ 25% total energy from protein) are recommended only in undernourished women to prevent stillbirth and SGA	0.8 g/kg/day (46 g/day)	0.8 increasing to 1.1 g/kg/day in 2nd half of pregnancy (71 g/day)
Total fibre ^4^	Fibre-rich diet may reduce preeclampsia and GDM but no specific recommendations are currently available; fibre supplements can be used to relieve constipation if diet modification is unsuccessful	14 g/1000 kcal (or 25 g/day)	14 g/1000 kcal or (or ~28 g/day to account for GWG)
Carbohydrates (GI and GL)	Low GL or GI diets may be beneficial for women at risk of GDM or LGA but can increase risk of SGA. No specific recommendations are currently available	130 g/day of carbohydrates	175 g/day of carbohydrates
Essential fatty acids ^4^ (linoleic acid [*n-6*] and α-linoleic acid [*n-3*])	*n-3* PUFAs may prevent preterm birth but can increase post-term birth and LGA. No specific recommendations are currently available	12 g/day (linoleic) 1.1 g/day (α-linoleic)	13 g/day (linoleic) 1.4 g/day (α-linoleic)
**Micronutrients**			
Folate/folic acid	Recommended (400 µg/day) from preconception until at least 12 weeks to prevent NTDs	400 µg/day	600 µg/day
Vitamin A	Not recommended except in areas with severe deficiency/night blindness	700 µg/day	770 µg/day
Thiamine (B_1_)	B-complex vitamins are not recommended to improve pregnancy outcomes until further evidence is available	1.1 mg/day	1.4 mg/day
Niacin (B_2_)		14 mg/day	18 mg/day
Riboflavin (B_3_)		1.1 mg/day	1.4 mg/day
Pyridoxine (B_6_)		1.3 mg/day	1.9 mg/day
Cyanocobalamin (B_12_)		2.4 µg/day	2.6 µg/day
Vitamin C	Not recommended until further evidence relating to safety and PROM is available	75 mg/day	85 mg/day
Vitamin E		15 mg/day	15 mg/day
Vitamin D ^4^	Not recommended for improving pregnancy outcomes but should be given to women with deficiency (200 IU/day)	5 µg/day	5 µg/day
Calcium ^4^	Recommended (1.5–2.0 g/day) to prevent hypertensive disorders in women with low dietary calcium intake or who are at high risk of hypertension	1 g/day	1 g/day
Iodine	Recommended only in women at high risk to prevent IDDs (i.e., in countries where < 20% of households have access to iodized salt)	150 µg/day	220–250 µg/day
Iron	Recommended (30–60 mg/day) to prevent maternal anaemia, puerperal sepsis, LBW and preterm birth	18 mg/day	27–60 mg/day
Zinc	Not recommended for improving pregnancy outcomes until more rigorous research is available	8 mg/day	11 mg/day
Alcohol	Not recommended during pregnancy until safe upper limits are established	NA	None
Caffeine	Reducing intake is recommended in women with high caffeine intake (> 300 mg/day) to prevent pregnancy loss and LBW infants	NA	<200 mg/day

^1^ Based on World Health Organization recommendations [79]; ^2^ dietary reference intakes derived from Institute of Medicine guidelines [18] and expressed as recommended dietary allowance (RDA) unless otherwise indicated; ^3^ EER = estimated energy requirement for adult women, reflecting the average energy intake predicted to maintain energy balance in a healthy adult of a defined age, weight, height and level of physical activity [18]; ^4^ values reflect average intakes (AI) as RDAs are not available. GWG, gestational weight gain; EER, estimated energy requirement; PA, physical activity; SGA/LGA, small-/large-for-gestational-age; GI, glycaemic index; GL, glycaemic load; GDM, gestational diabetes mellitus; PUFAs, polyunsaturated fatty acids; NTDs, neural tube defects; PROM, premature rupture of membranes; IDDs, iodine deficiency disorders; LBW, low birthweight; NA, not applicable.
